# Targeted metabolomic analysis of plasma metabolites in patients with coronary heart disease in southern China

**DOI:** 10.1097/MD.0000000000014309

**Published:** 2019-02-15

**Authors:** Zhixiong Zhong, Jing Liu, Qifeng Zhang, Wei Zhong, Bin Li, Cunren Li, Zhidong Liu, Min Yang, Pingsen Zhao

**Affiliations:** a Center for Cardiovascular Diseases, Meizhou People's Hospital (Huangtang Hospital), Meizhou Academy of Medical Sciences, Meizhou Hospital Affiliated to Sun Yat-sen University; b Guangdong Provincial Engineering and Technology Research Center for Molecular Diagnostics of Cardiovascular Diseases; c Meizhou Municipal Engineering and Technology Research Center for Molecular Diagnostics of Cardiovascular Diseases; d Clinical Core Laboratory, Meizhou People's Hospital (Huangtang Hospital), Meizhou Academy of Medical Sciences, Meizhou Hospital Affiliated to Sun Yat-sen University; e Center for Precision Medicine, Meizhou People's Hospital (Huangtang Hospital), Meizhou Academy of Medical Sciences, Meizhou Hospital Affiliated to Sun Yat-sen University; f Meizhou Municipal Engineering and Technology Research Center for Molecular Diagnostics of Major Genetic Disorders, Meizhou, PR China.

**Keywords:** biomarkers, coronary heart disease, metabolites, normal coronary artery, targeted metabolomic analysis

## Abstract

Coronary heart disease (CHD), one of the leading causes of death in the world, is a complex metabolic disorder due to genetic and environmental interactions. The potential mechanisms and diagnostic biomarkers for different types of coronary heart disease remain unclear. Metabolomics is increasingly considered to be a promising technology with the potential to identify metabolomic features in an attempt to distinguish the different stages of CHD.

We aimed to investigate serum metabolite profiling between CHD patients and normal coronary artery (NCA) subjects and identify metabolic biomarkers associated with CHD progression in an ethnic Hakka population in southern China.

Using a novel targeted metabolomics approach, we explored the metabolic characteristics of CHD patients. Blood samples from 302 patients with CHD and 59 NCA subjects were collected that analyses using targeted liquid-chromatography coupled with tandem mass spectrometry (LC-MS).

A total of 361 blood samples were determined using targeted LC-MS. Plasma concentrations for trimetlylamine oxide (TMAO), choline, creatinine, and carnitine were significantly higher in patients with CHD compared to the NCA cohort. Further, we observed that the concentration of the 4 metabolites were higher than that of the NCA group in any group of CHD, which including acute myocardial infarction (AMI), unstable angina (UA), and stable angina (SA). In addition, the diagnostic model was constructed based on the metabolites identified and the ROC curve of the NCA subjects and CHD patients were performed. For choline and creatinine, the AUCs ranged from 0.720 to 0.733. For TMAO and carnitine, the AUCs ranged from 0.568 to 0.600.

In conclusion, the current study illustrates the distribution of 4 metabolites between CHD patients and NCA subjects. Metabolomics analysis may yield novel predictive biomarkers that will potentially provide value for clinical diagnosis of CHD.

## 1 Introduction

Intestinal microflora has been recognized as a new endocrine organ, which plays an important role in regulating the metabolic function of host heart by regulating the blood level of bioactive metabolite.^[1,2]^ In recent years, studies have shown that gut microbes may promote the development of various diseases in animal models and patients, including diabetes and cardiovascular diseases.^[3]^ Recent studies by Koeth et al^[3]^ reported that microbial metabolism of dietary choline and phosphatidylcholine are associated with the pathogenesis of CVD (cardiovascular diseases) in both humans and mice. Choline, a compound containing trimethylamine and part of the head group of phosphatidylcholine, is important human nutrients that can be obtained from a variety of foods. Carnitine is also a nutrient, which plays a vital role in fatty acid metabolism. Choline and cartinine can be metabolized to trimethylamine (TMA) by the intestinal flora. TMA is rapidly oxidized by hepatic flavin monooxygenases to form trimethylamine *N*-oxide (TMAO), which is an intestinal dependent metabolite produced by TMA. TMAO is a small organic compound, which some types of foods (such as red meat, fish, poultry, and rays) are important direct sources of TMAO. The relationship between atherosclerosis and TMAO was confirmed by current metabonomics and model animal studies that is associated with cardiovascular risk.^[4]^ Impaired renal function is prevalent in the population.^[5–7]^ When serum creatinine is used as an indicator of impaired renal function in patients with cardiovascular disease, it was found to be an independent risk factor, which significantly increases morbidity and mortality.^[8]^ Also elevated plasma creatinine levels were correlated with an increased risk of heart attack and ischemic heart disease in patients with renal failure and diabetes.^[9]^

Although the treatment of coronary heart disease (CHD) has made considerable progress, it is still the main cause of mortality worldwide. It is well known that CHDs are associated with risk factors, which including obesity, hypertension, smoking, diabetes, hyperlipidemia, and age. Certain metabolites, such as cysteine, cholesterol, and triglycerides, have been strongly associated with CHD. Some researchers believe that there may be many other dysregulated metabolites that have not yet been described in addition these well-known associations.^[10–12]^ The rapid development of genomics, proteomics, metabolomics, and lipidomics has promoted many novel molecular biomarkers with potential clinical disease value. Among them, metabolomics, an effective method for detecting broad-spectrum small-molecule metabolites, has been increasingly applied to the studies of CHD, and has found novel metabolic biomarkers and relationship between the pathogenesis of CHD and metabolic.^[1,13,14]^ Previous metabolomics studies concerning CHD have shown that metabolites, such as TMAO and choline, are associated with an advanced cardiometabolic risk profile.^[15,16]^

In this study, in order to examine the plasma metabolomics characteristics of CHD and explore the potential biomarkers metabolism of CHD, we used targeted liquid chromatography coupled with LC-MS metabolomics analyze endogenous metabolites in plasma samples between NCA (without stenosis in coronary arteries) patients and CHD patients. Our findings may identify potential biomarkers for the differential diagnosis of CHD and insights into the underlying metabolic pathways that potentially involved in CHD pathogenesis

## 2 Methods

### 2.1 Patient population

The prospective cohort study was based on continuous recruitment of CHD subjects. Plasma samples of 361 subjects who underwent coronary angiography to evaluate CHD at Meizhou People's Hospital (Huangtang Hospital), Meizhou Academy of Medical Sciences, Meizhou Hospital Affiliated to Sun Yat-sen University between January 2017 and December 2017 were collected for our study. We excluded participants who had serious physical disease (such as a history of stroke or visceral bleeding disorders; severe liver disease or coagulation abnormalities; peripheral vascular disease, or infective endocarditis; chronic renal failure and chronic inflammatory history; malignant tumors; or any serious disease that might affect short-term prognosis), in comprehensible to the study, incomplete data on lipid files for patients, resulting in a cohort of 361 eligible participants. In addition, mild hypertension can be diagnosed when diastolic blood pressure values of 90 to 105 mm Hg diastolic blood pressure and/or 140 and 180 mm Hg systolic blood pressure are repeatedly measured over a period of at least 4 weeks and during this period without drug intervention.^[17]^ According to the guidelines for the prevention and the treatment of dyslipidemia in China, venous blood sample tests can be diagnosed as hyperlipidemia if they meet one of the following criteria: LDL-C > 3.64 mmol/L (140 mg/dL), HDL-C <0.91mmol/L (35 mg/dL), TG > 1.70 mmol/L (150 mg/dL), TC > 5.72 mmol/L (220 mg/dL).

To explore alterations in the plasma metabolites that may be associated with CHD, 2 independent case–control groups were constructed based on strict coronary angiographic evidence: one group consisted of 302 CHD subjects (at least 1 major coronary artery with ≥50% stenosis), and one group consisted of 59 NCA (without stenosis in coronary arteries). Coronary heart disease is divided into acute myocardial infarction (AMI), unstable angina pectoris (UA) or stable angina (SA) according to the severity of the disease.

A total of 361 CHD patients at Meizhou People's Hospital (Huangtang Hospital), Meizhou Academy of Medical Sciences, Meizhou Hospital Affiliated to Sun Yat-sen University, were eligible for this study. The present study was performed in accordance with the ethics standards laid down in the updated version of the 1964 Declaration of Helsinki and was approved by the Ethics Committees of Meizhou People's Hospital (Huangtang Hospital), Meizhou Academy of Medical Sciences, Meizhou Hospital Affiliated to Sun Yat-sen University, Guangdong province, China. All patients had signed informed consent.

### 2.2 Preparation of standards, internal standards, and quality control samples

To find the linear range, LOD and lower LOQ (LLOQ), standards of 7 different concentrations were made ranging over 3 orders of magnitude. The highest concentration calibration standard was first prepared in acetonitrile containing 1% formic acid (at a final concentration of 40 μM for choline, 1 μM for TMAO, and 20 μM for creatinine, 20 μM for L-carnitine). The remaining calibration standards were then prepared by twice or 2 point fivefold serial dilution of the highest concentration standard. Accurately weigh the appropriate amount of choline, betaine, TMAO, creatinine, and L-carnitine standards, and the internal standard working solution was prepared by mixing 4 standards into acetonitrile with 1% formic acid to final concentrations of 0.5, 0.25, 50, and 1 μg/mL for choline-d9, TMAO-d9, and creatinine-d3, L-carnitine-d3, respectively. Finally, it was stored at –20°C in small aliquots.

Three concentration levels of quality control (QC) samples were prepared by spiking pooled 20 μl human plasma sample with stock solutions of choline, TMAO, creatinine and L-carnitine to low (LQC; the pooled plasma spiked with 0.8 μM of choline, 20 ng/mL of TMAO, 400 ng/mL of creatinine, and 400 ng/mL of L-carnitine), medium (MQC; the pooled plasma spiked with 4 μM M of choline, 100 ng/mL of TMAO, 2000 ng/mL of creatinine, and 2000 ng/mL of L-carnitine) and high (HQC; the pooled plasma spiked with 20 μM of choline, 500 ng/mL of TMAO, 10000 ng/mL of creatinine, and 10000 ng/mL of L-carnitine). The QC samples were then divided into polypropylene tubes and stored in the freezer at −80°C. These quality control samples were used for method validation.^[18,19]^

### 2.3 Serum collection and preparation

Nonfasting plasma samples were collected in sterile EDTA containers (BD Microtainer) before the coronary angiography and stored at 4°C, and then centrifuged a 2300×*g* for 10 minutes. The supernatants plasma was stored at −80°C. Before the metabonomics analysis, plasma samples were thawed at 4°C on the ice. Around 20 μL of each sample or standard solution were precipitated by adding 10 μl internal standard solution, then add 750 μL of 1% formic acid-acetonitrile solution. The samples were thoroughly mixed and centrifuged for 10 minutes at 12000 rpm, and the supernatants were subjected to LC/MS analysis.

### 2.4 Metabolomic analysis by LC/MS

The plasma samples were randomly injected for LC/MS analysis in both the electrospray positive and positive ionization mode. The plasma samples concentrations of TMAO, choline, creatinine, and cartinine were quantified by using Acquity UPLC system (Waters Corporation; Milford, MA), coupled with online electrospray ionization tandem mass spectrometry (AB 4000 Triple Quadrupole Mass Spectrometer (AB 4000). Chromatographic separation was achieved using an ACQUITY UPLC BEH C18 (100 × 2.1 mm, 1.7 μm, Waters) column maintained at 40°C with an injection velum of 5 μL. The auto-sampler temperature was maintained at 4°C. Solvents were delivered at a flow rate 0.4 mL/min, using water (solvent A) and acetonitrile (solvent B) both containing 0.1% formic acid. The elution conditions were 0 to 1 minute, 80% A; 1 to 2 minutes, 80 to 70% A; 2 to 2.5 minutes, 70% A; 2.5 to 3 minutes, 70 to 50% 3.5 to 4 minutes, 50 to 80% A; 4 to 6 minutes, 80% A.

### 2.5 Method validation

Four analysis runs were performed on different days (day 1, day 2, day 3, and day 4) to assess accuracy. Calibration standards are repeated at the beginning and end of each batch. The linearity of each substance is good in the range of concentration. The intra-day and inter-day precision are <15%, the recovery is between 85% and 115%, and the stability deviation is within ±15%. The method meets the requirements of sample analysis.

### 2.6 Statistical analysis

Statistical analysis of the data was performed by using SPSS 21.0 software (SPSS, Inc., Chicago, IL). Categorical variables were analyzed using chi-square test and continuous variables were tested by Kruskal–Wallis test or one-way ANOVA. Receiver operating characteristic curve^[20]^ analysis was used to determine the clinical utility of each metabolite. A probability value of *P < *.05 was considered significant for this study.

## 3 Results

### 3.1 Patient characteristics in CHD cohorts

**Figure 1:**
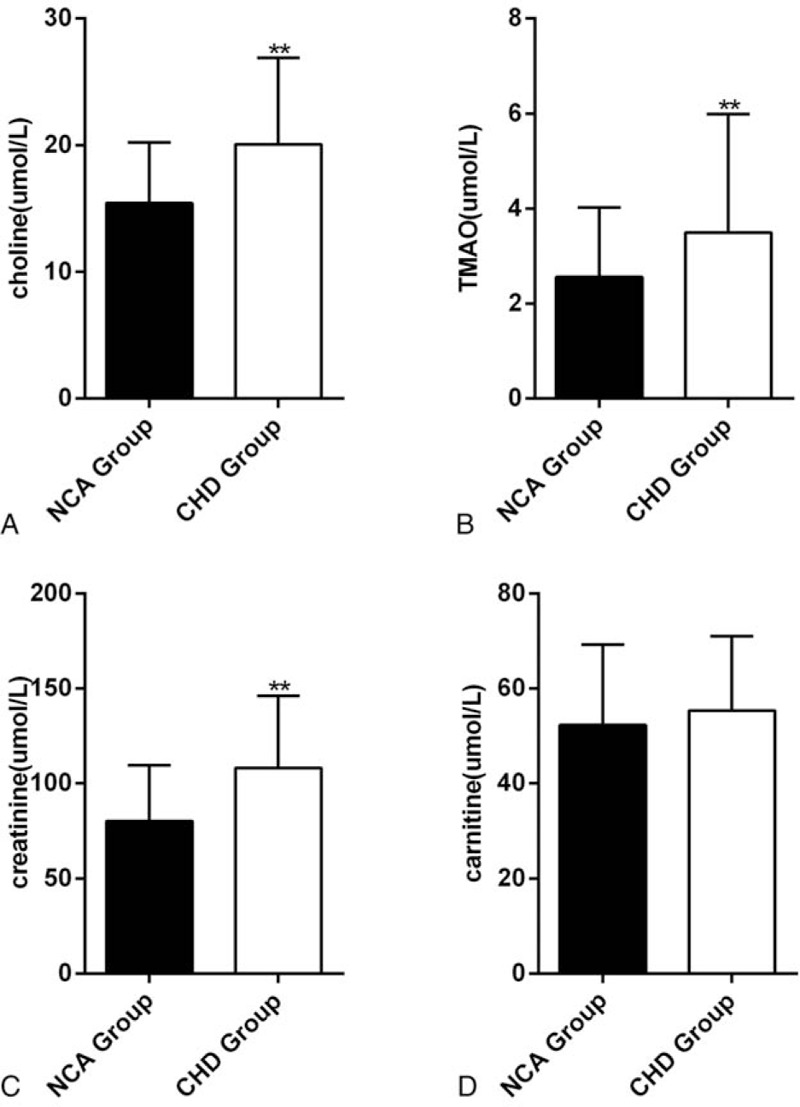
Comparisons in choline, TMAO, creatinine and cartinine levels between individuals with coronary heart disease (n = 302) and NCA (n = 59) for (A), (B), (C), (D), ^∗∗^*P* < .01. NCA = normal coronary artery, TMAO = trimetlylamine oxide.

A total of 361 subjects were recruited in our study cohort (59 subjects in the NCA group and 302 patients in the CHD group), which 140 patients were excluded with unavailable diagnostic data in Figure 1. Table 1 shows the baseline features of 361 participants. The average age of CHD patients was 65.86 ± 10.71 years and that of NCA subjects were 61.20 ± 8.98 years. Additionally, 78.4% of patients were males. In our study cohort, compare with NCA group, CHD subjects were more likely to be older, with more history of diabetes mellitus, smoker, prior history of CAD, drinking, hypertension, hyperlipidemia and significantly increased levels of TG (*P < *.038), HDL (*P < *.001).

**Table 1:**
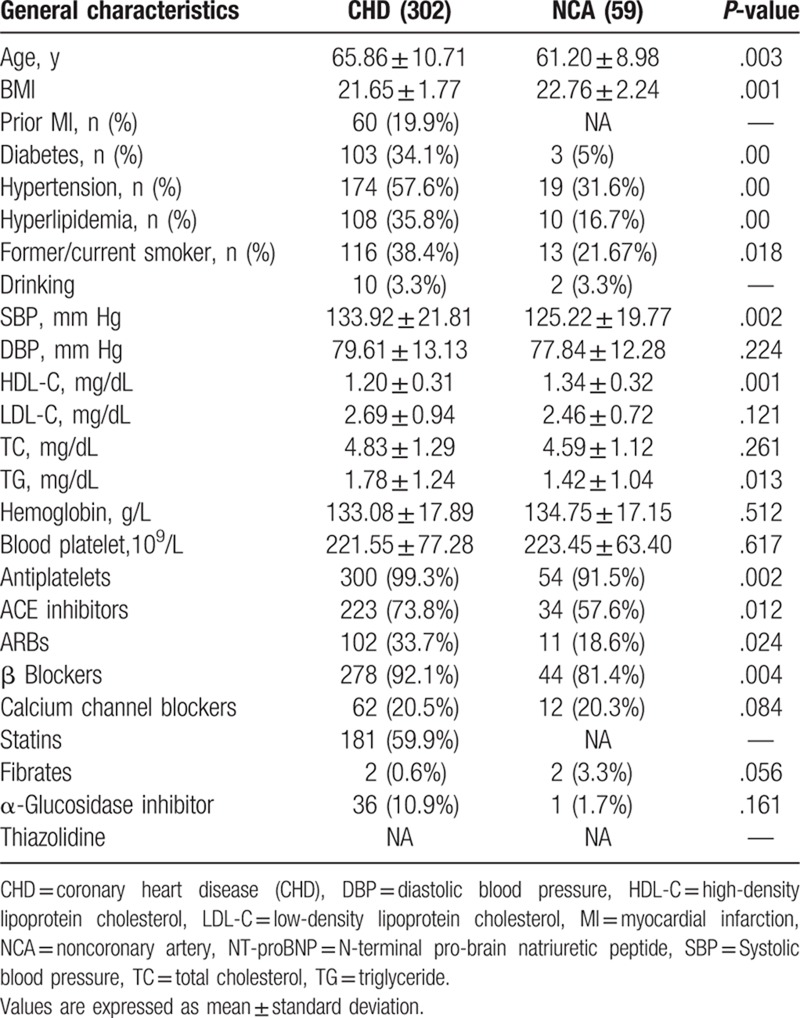
Baseline clinical characteristics10^9.^

### 3.2 Analysis of metabolomics characteristics of plasma samples between NCA subjects and CHD patients

**Figure 2:**
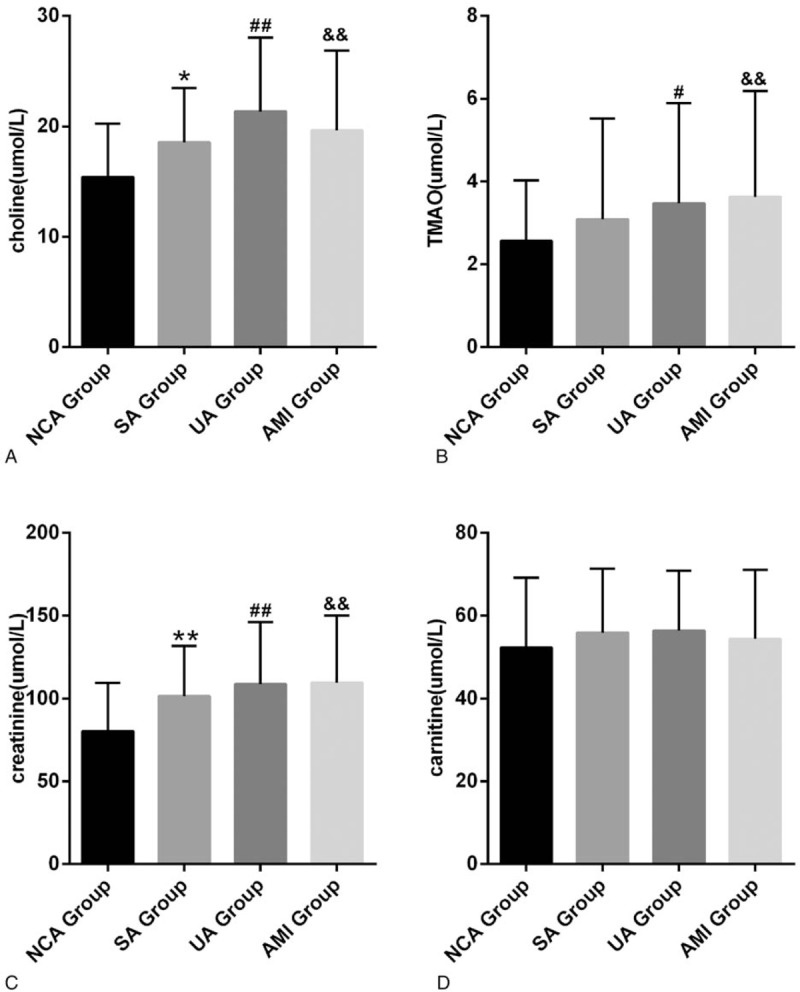
Choline, TMAO, creatinine and cartinine levels in different groups. (A) Comparison between NCA group and SA, UA, AMI groups for choline, respectively, ^∗^*P* < .05, ^*∗∗*^*P* < .01, ^#^*P* < .05, ^##^*P* < .01, ^*&*^*P* < .01, ^*&&*^*P* < .01. (B) Comparison between NCA group and SA, UA, AMI groups for TMAO, respectively, ^#^*P* < .05, ^##^*P* < .01, ^*&*^*P* < .01,^*&&*^*P* < .01. (C) For creatinine, comparison between NCA group and SA, UA, AMI groups for TMAO, respectively, ^∗∗^*P* < .01, ^##^*P* < .01,^*&&*^*P* < .01.(D) There have no significantly difference between NCA group and SA, UA, AMI groups for carnitine, respectively. AMI = acute myocardial infarction, NCA = normal coronary artery, SA = stable angina, TMAO = trimetlylamine oxide, UA = unstable angina.

We firstly performed a cross-sectional comparison of the concentrations of the 4 metabolites in the NCA and ACS groups, respectively. The results shown that the plasma concentrations for TMAO, choline, creanitine, and cartinine were each significantly higher in patients with CHD compared to the NCA cohort (Fig. 1). Further, to determine metabolomics characteristics for CHD patients with different disease severity, we divided CHD patients into three groups which including SA, UA, and AMI group. We observed that the concentration of the 4 metabolites were higher than that of the NCA group in any 3 groups (Fig. 2). These results demonstrated the existence of different plasma metabolites signatures between NCA subjects and CHD patients. The above results are consistent with those observed of other researchers.

### 3.3 Diagnostic accuracy of the differential metabolite

**Figure 3:**
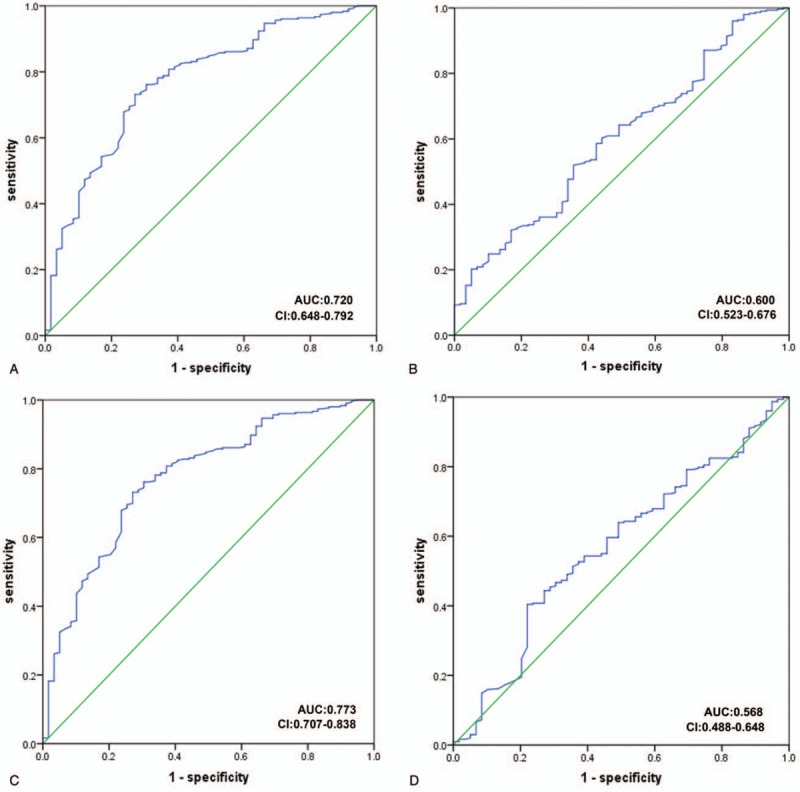
Receiver operating characteristics (ROC) curve model of metabolites to discriminate CHD patients from NCA subjects. (A: choline; B: TMAO; C: creatinine; D: carnitine).

To test the clinical diagnostic of differential metabolite, ROC curve of the NCA subjects and CHD patients were performed. For choline and creatinine, the AUCs ranged from 0.720 to 0.733 (Fig. 2A and C). For TMAO and carnitine, The AUCs ranged from 0.568–0.600 (Fig. 2B and D). However, these metabolites only moderate difference between NCA patients and CHD patients with an AUC range from 0.568 to 0.733, as shown in Figure 3.

## 4 Discussion

CHD, one of the leading causes of death in the world, is a complex metabolic disorder due to genetic and environmental interactions.^[21–23]^ Metabolomics, a powerful technical system that studies the entire metabolic pathway of a particular biological system. This technology has been increasingly used to identify metabolic biomarkers and improve clinical diagnosis and treatment of diseases.^[24,25]^ Recent studies have shown that changes in metabolites reflect the onset and progression of CHD.^[13,26]^ Some metabolic biomarkers identified by metabolomics have the potential to provide diagnostic and predictive value for CHD.^[25,27–30]^ However, a large number of validations are still required before these putative “biomarkers” are used in the diagnosis of CHD due to the in-depth study of the mechanism of targeted metabolomics.^[31]^

In this study, we performed a targeted metabolomics assessment of plasma from CHD patients and NCA and identified metabolic features from these 2 sets of metabolites. The plasma concentrations for TMAO, choline, creatinine, and cartinine were each significantly higher in patients with CHD compared to the NCA cohort. These results were consistent with the current clinical metabolomics. Then, we performed ROC curve analysis on the 4 metabolites. Surprisingly, choline and creatinine metabolite concentrations can be significantly discrimination between CHD and NCA, suggesting that it may become biomarkers of CHD in future clinical studies.

Choline is a component of phosphatidylcholine, which is very important to the structural integrity of cell membrane, the synthesis of acetylcholine, the signaling of cell membrane, and the metabolism of methyl group.^[32]^ The last function occurs through betaine, which is a choline metabolite. To our knowledge, plasma choline has not previously been explored as predictors of CHD or all-cause mortality in CHD patients. While Wang et al^[1]^ reported the relationship between high-dose dietary choline and increased atherosclerotic plaque formation in mice, possibly due to increased TMAO production. Every year, about 20 million patients are affected by sudden fatal or nonfatal cardiac events (ACS or CHD, including AMI and/or sudden cardiac death) and plaque vulnerability is a major underlying pathology.^[33]^ Danne and Möckel^[33]^ found that elevated choline in whole blood and plasma was associated with plaque instability in acute coronary syndrome. A very interesting imaging study suggests that choline may become a comprehensive biomarker of atherosclerotic plaque vulnerability. Meanwhile, other investigators have also found that choline may be useful for early risk stratification in patients with ACS, especially when troponin is negative on admission.^[34]^ In a recent prospective study of patients undergoing selective coronary angiography, the results showed that TMAO plasma levels were associated with major cardiovascular events during a 3-year follow-up;^[35]^ The data of dietary intake or plasma choline levels were not provided. Even 2 large prospective studies in the general population have shown that plasma choline levels are not associated with cardiovascular risk.^[36]^ Therefore, there are some inconsistencies in the literature on the relationship between choline and CVD. However, little studies indicated diagnostic analysis of choline.^[37]^ Our results show ROC curve analysis for CHD demonstrated an AUC of 0.720 (95% CI 0.648–0.792) for choline. Although our study suggests that a single metabolite may not be used as a biomarker to distinguish NCA from CHD, a combination with other clinical biochemical markers may provide clinical value for diagnosis, and we will continue to explore the link between choline and cardiovascular disease on the basis of these results.

Creatinine is a widely used renal function biomarker and is a metabolic waste released by muscle tissue during muscle contraction.^[9]^ Epidemiological studies have shown that elevated creatinine levels are correlated with an increased risk of cardiovascular disease, especially are associated with hypertension and diabetes.^[38,39]^ At present, different survival rates of different creatinine levels are not shown in the general population study. In our results in Table 1, we can conclude that creatinine was higher in patients with CHD compared to the NCA cohort, which CHD patients have more diabetes and hypertension. Creatinine may contribute to cardiovascular disease by accelerating atherosclerosis through several mechanisms/pathways, which including the extent and number of atherosclerotic lesions increased, increase in number and of low-density lipoprotein particles.^[40,41]^ Furthermore, high levels of plasma creatinine are associated with high triglycerides levels and low HDL cholesterol levels, causing an atherogenic lipid profile.^[9]^ So far, there was a study that confirmed creatinine is a biomarker of increased risk of myocardial infarction, ischemic heart disease, and early death in the general population.^[9]^ Therefore, we explore whether creatinine can be used as a marker for CHD. Our results show ROC curve analysis for CHD demonstrated an AUC of 0.773 (95% CI 0.648–0.792) for creatinine, which may provide new pathways for clinical diagnosis.

Many studies have shown that TMAO is a strong predictor of the risk of coronary artery disease, and then animal studies reveal the causal relationship between TMAO and atherosclerosis. However, group differentiation was poor although TMAO and carnitine were significantly higher in patients with CHD according to these abnormal metabolites.

Some of the limitations of our study are worth mentioning. Firstly, we enrolled patients based on clinical diagnosis, however, the number of patients enrolled in the study was relatively small due to limited funding, and patients of follow-up study is ongoing. Secondly, these metabolites were only tested at one time point, we are unable to assess the prognostic role of metabolites in CHD patients over time. Finally, our results are based on the Hakka population, which may not apply to individuals of different demographics.

## 5 Conclusion

We have identified metabolites in the NCA and CHD groups using targeted LC-MS metabolomic analysis. Significant differences in metabolites may be involved in the occurrence and development of atherosclerosis in CHD patients. ROC curve analysis shows that choline and creatinine may yield novel predictive biomarkers that will potentially provide value for clinical diagnosis of CHD. However, these results are preliminary studies that require further confirmation of the results and clarify their specific mechanisms in CHD.

## Acknowledgments

The author would like to thank other colleagues whom were not listed in the authorship of Center for Cardiovascular Diseases, Clinical Core Laboratory and Center for Precision Medicine, Meizhou People's Hospital (Huangtang Hospital), Meizhou Academy of Medical Sciences, Meizhou Hospital Affiliated to Sun Yat-sen University for their helpful comments on the manuscript.

## Author contributions

Pingsen Zhao conceived and designed the experiments; Zhixiong Zhong, Jing Liu and Pingsen Zhao recruited subjects and collected clinical data. Jing Liu conducted the laboratory testing. Qifeng Zhang, Wei Zhong, Bin Li, Cunren Li, Zhidong Liu and Min Yang helped to analyze the data. Pingsen Zhao, Zhixiong Zhong and Jing Liu prepare the manuscript. Pingsen Zhao and Zhixiong Zhong reviewed the manuscript.

**Conceptualization:** Pingsen Zhao.

**Data curation:** Zhixiong Zhong, Jing Liu, Qifeng Zhang, Pingsen Zhao.

**Formal analysis:** Zhixiong Zhong, Jing Liu, Qifeng Zhang, Wei Zhong, Bin Li, Cunren Li, Zhidong Liu, Min Yang, Pingsen Zhao.

**Funding acquisition:** Pingsen Zhao.

**Investigation:** Zhixiong Zhong, Jing Liu, Pingsen Zhao.

**Methodology:** Zhixiong Zhong, Jing Liu, Wei Zhong, Bin Li, Cunren Li, Zhidong Liu, Min Yang, Pingsen Zhao.

**Project administration:** Pingsen Zhao.

**Resources:** Zhixiong Zhong, Jing Liu, Qifeng Zhang, Wei Zhong, Bin Li, Cunren Li, Zhidong Liu, Min Yang, Pingsen Zhao.

**Software:** Zhixiong Zhong, Pingsen Zhao.

**Supervision:** Pingsen Zhao.

**Validation:** Pingsen Zhao.

**Visualization:** Pingsen Zhao.

**Writing – original draft:** Zhixiong Zhong, Jing Liu, Pingsen Zhao.

**Writing – review & editing:** Pingsen Zhao.

## References

[1] Wang Z Klipfell E Bennett BJ Gut flora metabolism of phosphatidylcholine promotes cardiovascular disease. Nature 2011;472:57–63. 21475195 10.1038/nature09922 PMC3086762

[2] Mente A Chalcraft K Ak H The relationship between trimethylamine-N-oxide and prevalent cardiovascular disease in a multiethnic population living in Canada. Can J Cardiol 2015;31:1189–94. 26239008 10.1016/j.cjca.2015.06.016

[3] Koeth RA Wang Z Levison BS Intestinal microbiota metabolism of l-carnitine, a nutrient in red meat, promotes atherosclerosis. Nat Med 2013;19:576–85. 23563705 10.1038/nm.3145 PMC3650111

[4] MT V A. R A. M Trimethylamine *N*-oxide: the good, the bad and the unknown. Toxins 2016;8:326. 10.3390/toxins8110326 PMC5127123 27834801

[5] Shlipak MG Heidenreich PA Noguchi H Association of renal insufficiency with treatment and outcomes after myocardial infarction in elderly patients. Ann Int Med 2002;137:555. 12353942 10.7326/0003-4819-137-7-200210010-00006

[6] Gottdiener JS Arnold AM Aurigemma GP Predictors of congestive heart failure in the elderly: the cardiovascular health study ☆. J Am Coll Cardiol 2000;35:1628–37. 10807470 10.1016/s0735-1097(00)00582-9

[7] Beilby J Divitini ML Knuiman MW Comparison of cystatin C and creatinine as predictors of cardiovascular events in a community-based elderly population. Clin Chem 2010;56:799–804. 20207770 10.1373/clinchem.2009.135962

[8] Shlipak MG Simon JA Grady D Renal insufficiency and cardiovascular events in postmenopausal women with coronary heart disease. J Am Coll Cardiol 2001;38:705–11. 11527621 10.1016/s0735-1097(01)01450-4

[9] Sibilitz KL Benn M Nordestgaard BG Creatinine, eGFR and association with myocardial infarction, ischemic heart disease and early death in the general population. Atherosclerosis 2014;237:67–75. 25222342 10.1016/j.atherosclerosis.2014.08.040

[10] Li Y Zhang D He Y Investigation of novel metabolites potentially involved in the pathogenesis of coronary heart disease using a UHPLC-QTOF/MS-based metabolomics approach. Sci Rep 2017;7:15357. 29127404 10.1038/s41598-017-15737-3 PMC5681629

[11] Nygã¥Rd O Nordrehaug JE Refsum H Plasma homocysteine levels and mortality in patients with coronary artery disease. N Engl J Med 1997;337:230–6. 9227928 10.1056/NEJM199707243370403

[12] Rubins HB Triglycerides and coronary heart disease: implications of recent clinical trials. J Cardiovasc Risk 2000;7:339–45. 11143764 10.1177/204748730000700507

[13] Shah SH Bain JR Muehlbauer MJ Association of a peripheral blood metabolic profile with coronary artery disease and risk of subsequent cardiovascular events. Circ Cardiovasc Genet 2010;3:207–14. 20173117 10.1161/CIRCGENETICS.109.852814

[14] Magnusson M Lewis GD Ericson U A diabetes-predictive amino acid score and future cardiovascular disease. Eur Heart J 2013;34:1982–9. 23242195 10.1093/eurheartj/ehs424 PMC3703309

[15] Bidulescu A Chambless LE Siegariz AM Usual choline and betaine dietary intake and incident coronary heart disease: the Atherosclerosis Risk in Communities (ARIC) study. BMC Cardiovasc Disord 2007;7:1–8. 17629908 10.1186/1471-2261-7-20 PMC1934379

[16] Kaysen GA Johansen KL Chertow GM Associations of trimethylamine N-oxide with nutritional and inflammatory biomarkers and cardiovascular outcomes in patients new to dialysis. J Renal Nutr 2015;25:351–6. 10.1053/j.jrn.2015.02.006 PMC4469547 25802017

[17] Group BMJP. Summary of 1993 World Health Organisation-International Society of Hypertension guidelines for the management of mild hypertension. Subcommittee of WHO/ISH Mild Hypertension Liaison committee. BMJ 1993;307:1541–6. 8274926 10.1136/bmj.307.6918.1541 PMC1679536

[18] Grinberga S Dambrova M Latkovskis G Determination of trimethylamine-N-oxide in combination with l-carnitine and γ-butyrobetaine in human plasma by UPLC/MS/MS. Biomed Chromatogr 2015;29:1670–4. 25873316 10.1002/bmc.3477

[19] Zhao X Zeisel SH Zhang S Rapid LC-MRM-MS assay for simultaneous quantification of choline, betaine, trimethylamine, trimethylamine N-oxide, and creatinine in human plasma and urine. Electrophoresis 2015;36:2207–14. 26081221 10.1002/elps.201500055

[20] Aynacioglu AS Brockmoller J Bauer S Frequency of cytochrome P450 CYP2C9 variants in a Turkish population and functional relevance for phenytoin. Br J Clin Pharmacol 1999;48:409–15. 10510154 10.1046/j.1365-2125.1999.00012.x PMC2014334

[21] Lu J Chen B Chen T Comprehensive metabolomics identified lipid peroxidation as a prominent feature in human plasma of patients with coronary heart diseases. Redox Biol 2017;12:899. 28472752 10.1016/j.redox.2017.04.032 PMC5415551

[22] Słomka A Piekuś A Kowalewski M Assessment of the procoagulant activity of microparticles and the protein z system in patients undergoing off-pump coronary artery bypass surgery. Angiology 2017;69:3319717706616. 10.1177/0003319717706616 28464697

[23] Słomka A Korbal P Piekuś A Plasma levels of the A subunit of factor XIII in patients undergoing off-pump coronary artery bypass surgery. Pol Arch Intern Med 2017;127:550–3. 28817543 10.20452/pamw.4076

[24] Rhee EP Gerszten RE Metabolomics and cardiovascular biomarker discovery. Clin Chem 2012;58:139. 22110018 10.1373/clinchem.2011.169573 PMC4402975

[25] Fan Y Li Y Chen Y Comprehensive metabolomic characterization of coronary artery diseases. Sci Found China 2016;68:1281. 10.1016/j.jacc.2016.06.044 27634119

[26] Mã MNGL Ruiz-Canela M Hruby A Intervention trials with the mediterranean diet in cardiovascular prevention: understanding potential mechanisms through metabolomic profiling. J Nutrit 2016;146:913S. 10.3945/jn.115.219147 PMC4807639 26962184

[27] Liu X Gao J Chen J Identification of metabolic biomarkers in patients with type 2 diabetic coronary heart diseases based on metabolomic approach. Sci Rep 2016;6:30785. 27470195 10.1038/srep30785 PMC4965763

[28] Wei G Jiang C Liu Y Quantitative metabolomic profiling of plasma, urine, and liver extracts by 1H NMR spectroscopy characterizes different stages of atherosclerosis in hamsters. J Proteome Res 2016;15:3500. 27570155 10.1021/acs.jproteome.6b00179

[29] Garcíafontana B Moralessantana S Díaz NC Metabolomic profile related to cardiovascular disease in patients with type 2 diabetes mellitus: A pilot study. Talanta 2016;148:135–43. 26653434 10.1016/j.talanta.2015.10.070

[30] Barallobrebarreiro J Chung YL Mayr M Proteomics and metabolomics for mechanistic insights and biomarker discovery in cardiovascular disease. Rev Esp Cardiol 2013;66:657–61. 24776335 10.1016/j.rec.2013.04.009

[31] Baig F Pechlaner R Mayr M Caveats of untargeted metabolomics for biomarker discovery. J Am Coll Cardiol 2016;68:1294. 27634120 10.1016/j.jacc.2016.05.098

[32] Ierardi E Sorrentino C Principi M Intestinal microbial metabolism of phosphatidylcholine: a novel insight in the cardiovascular risk scenario. Hepatobiliary Surg Nutr 2015;4:289. 26312245 10.3978/j.issn.2304-3881.2015.02.01 PMC4526767

[33] Danne O Möckel M Choline in acute coronary syndrome: an emerging biomarker with implications for the integrated assessment of plaque vulnerability. Expert Rev Mol Diagn 2010;10:159–71. 20214535 10.1586/erm.10.2

[34] Danne O Möckel M Lueders C Prognostic implications of elevated whole blood choline levels in acute coronary syndromes ☆. Am J Cardiol 2003;91:1060–7. 12714147 10.1016/s0002-9149(03)00149-8

[35] Tang WHW Wang Z Levison BS Intestinal microbial metabolism of phosphatidylcholine and cardiovascular risk. N Engl J Med 2013;368:1575–84. 23614584 10.1056/NEJMoa1109400 PMC3701945

[36] Dalmeijer GW Olthof MR Verhoef P Prospective study on dietary intakes of folate, betaine, and choline and cardiovascular disease risk in women. Eur J Clin Nutr 2008;62:386–94. 17375117 10.1038/sj.ejcn.1602725

[37] Schartum-Hansen H Pedersen ER Svingen GF Plasma choline, smoking, and long-term prognosis in patients with stable angina pectoris. Eur J Prev Cardiol 2015;22:606–14. 24595862 10.1177/2047487314524867

[38] Culleton BF Larson MG Evans JC Prevalence and correlates of elevated serum creatinine levels: the Framingham Heart Study. Arch Intern Med 1999;159:1785–90. 10448783 10.1001/archinte.159.15.1785

[39] Wannamethee SG Shaper AG Perry IJ Serum creatinine concentration and risk of cardiovascular disease: a possible marker for increased risk of stroke. Stroke 1997;28:557–63. 9056611 10.1161/01.str.28.3.557

[40] Bro S Borup R Andersen CB Uremia-specific effects in the arterial media during development of uremic atherosclerosis in apolipoprotein E-deficient mice. Arterioscler Thromb Vasc Biol 2006;26:570–5. 16373611 10.1161/01.ATV.0000201060.47945.cb

[41] Simolin MA Pedersen TX Bro S ACE inhibition attenuates uremia-induced aortic valve thickening in a novel mouse model. BMC Cardiovasc Disord 2009;9:1–0. 19257900 10.1186/1471-2261-9-10 PMC2663538

